# Profound Trunnion Wear Resulting in Femoral Head-Neck Dissociation in Total Hip Arthroplasty

**DOI:** 10.1155/2018/1534572

**Published:** 2018-08-23

**Authors:** Saif Shamshoon, Patrick Thornley, Justin de Beer

**Affiliations:** ^1^Faculty of Health Sciences, Department of Surgery, McMaster University, Hamilton, Ontario, Canada; ^2^Division of Orthopaedic Surgery, McMaster University, Hamilton, Ontario, Canada; ^3^Hamilton Health Sciences, Juravinski Hospital, Hamilton, Ontario, Canada; ^4^Hamilton Arthroplasty Group, Hamilton Health Sciences, Juravinski Hospital, Hamilton, Ontario, Canada

## Abstract

We describe a case of aseptic failure with profound femoral stem trunnion wear and femoral head dissociation nine years after initial primary total hip arthroplasty (THA) with the Stryker Accolade total hip arthroplasty system. Current guidelines for postoperative care and follow-up after THAs as potential intervention points for early detection of prosthetic joint failure are also reviewed.

## 1. Introduction

Trunnion wear in the setting of total hip arthroplasty (THA) continues to be an important cause of prosthesis failure, accounting for up to 3% of THA revision procedures [1]. Though the causes of trunnion damage are unknown, it is thought to be due to a combination of motion of the prosthetic head on the neck, as well as electrochemical corrosion. Additionally, the geometry of different implant designs has been noted to potentially increase the risk of trunnion failure [1].

While metallosis has classically been more frequently documented in the setting of metal-on-metal prosthesis, there have been reports of elevated blood concentrations of metallic ions in association with trunnion wear in some metal-on-polyethylene THAs [2, 3]. The following case describes a patient who presented with femoral stem-head dissociation and associated metallosis nine years after initial right total hip arthroplasty using the Stryker Accolade press-fit metal-on-polyethylene total hip arthroplasty system.

## 2. Case Report

An 88-year-old man, BMI 24, presented to the clinic with complaints of pain and a cracking sensation in his right hip, nine years post primary right THA. His previous medical history was significant for coronary artery disease, myocardial infarction, hypertension, chronic kidney disease, and gastroesophageal reflux disease. His medications included aspirin, metoprolol, ramipril, simvastatin, and ranitidine.

**Figure 1:**
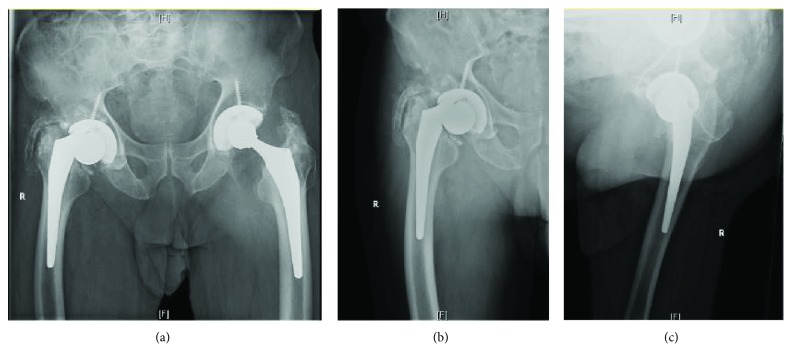
(a–c) Radiographic images illustrating marked discontinuity of the femoral head and neck, as well as shortening of the right hip prosthesis in what was initially thought to be a trunnion fracture.

During the initial procedure, the primary components utilized an Accolade #3 stem with a 127-degree neck-shaft angle and +5 36 mm L-fit head. Postoperative course for this patient was uncomplicated. After routine follow-up, at the one-year postoperative mark, the patient was subsequently lost to follow-up. Nine years after the index procedure, the patient returned to the clinic complaining of pain and “cracking” in the right hip for approximately six months. The patient denied any history of injury as well as any subjective infectious symptoms. Radiographic images of the right hip showed an apparent trunnion fracture with significant asymmetric wear of the polyethylene liner within the acetabular component (Figure 1).

Preoperative bloodwork revealed normal leukocytes and normal-range C-reactive protein (CRP) and erythrocyte sedimentation rate (ESR). Thus, the patient was consented for revision THA for a presumed trunnion fracture.

**Figure 2:**
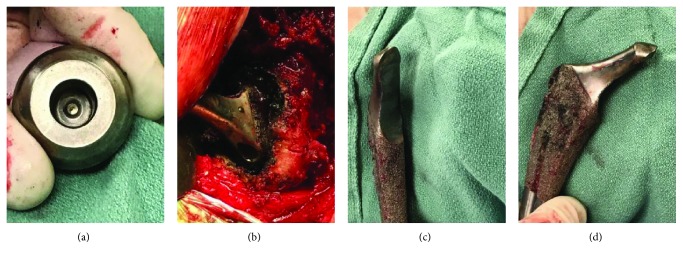
(a) Femoral head showing no gross signs of wear. (b) Metal-stained debris and granulation tissue widespread within the surgical site. (c, d) Significant trunnion wear along the femoral stem of the primary hardware.

**Figure 3:**
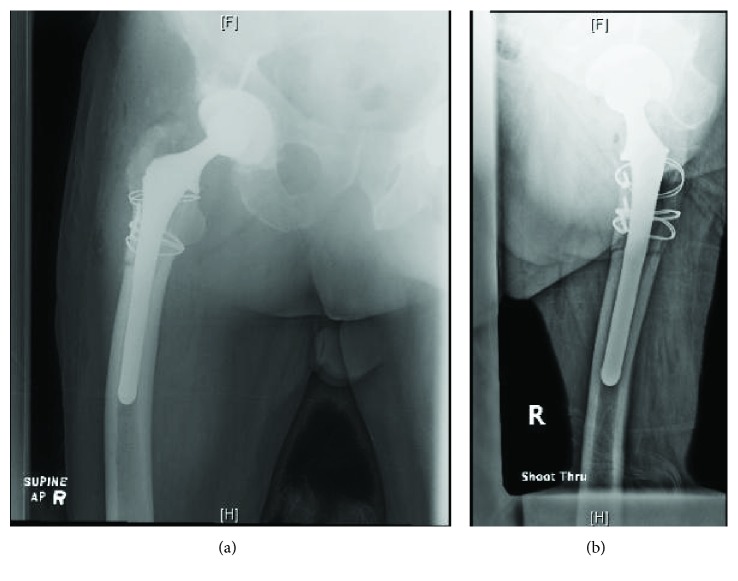
(a) Anterior-posterior radiograph demonstrating stable fixation after revision surgery. (b) Lateral radiograph demonstrating the same postrevision fixation.

Intraoperative assessment revealed marked heterotopic ossification along the greater trochanter of the right femur, extending into the gluteus maximus, which was excised and debrided thoroughly. Though the femoral head was intact and showed no gross signs of wear (Figure 2(a)), there was complete dissociation from the femoral stem. There were also significant signs of metallosis with metal-stained debris and granulation tissue (Figure 2(b)), which extended deep to the margins surrounding the acetabular shell. Interestingly, the femoral stem demonstrated significant medial trunnion wear (see Figures 2(c) and 2(d)), though no true trunnion fracture was noted, contrary to the initial preoperative radiographic assessment. Stem removal was facilitated by an extended trochanteric osteotomy, and definitive reconstruction was performed with a long cementless femoral stem and Luque wire fixation of the osteotomy. Intraoperative assessment demonstrated a stable final construct (Figures 3(a) and 3(b)). The patient had an uneventful postoperative recovery and at the time of the 6-week follow-up had completed his outpatient rehab protocol with excellent effect.

## 3. Discussion

While there are obvious advantages to modular hip prostheses, the potential for trunnionosis in a small patient subset is a potential cause of failure [4]. The incidence of trunnionosis has been well documented in the setting of metal-on-metal THAs but there are fewer accounts of these negative outcomes in metal-on-polyethylene THAs. While there are limited reports of trunnion wear using the Accolade total hip arthroplasty system, to the best knowledge of the research team, no reports exist demonstrating trunnion wear to such a significant level (Figure 2(c)) as that seen in our case [3, 5, 6]. This degree of trunnion wear may be attributable to the fact that the trunnion was lodged within the acetabular component of the prosthesis (Figures 1(a) and 1(b)) without the patient presenting to the clinic until at least six months after symptomatic hardware onset. While there have been attempts made to predict patients at most risk for trunnionosis after THA, reports suggest that males, with BMI > 30 kg/m^2^, are at greatest risk for trunnionosis [1]. Additionally, it has been repeatedly demonstrated that the use of larger-diameter femoral heads increases the effective horizontal lever arm, imparting greater torsional forces at the head-neck junction thus increasing the risk of trunnionosis [7–10]. Specifically, both Matthies et al. and Dyrkacz et al. have demonstrated that prosthetic femoral heads equal to or greater than 36 mm in diameter result in worse outcomes with respect to trunnion corrosion [9, 10].

Morlock et al. further speculated that taper and implant designs, as well as the loading situation in the patient and the assembly situation by the surgeon, play critical roles in the phenomenon of trunnion wear [11]. In our case, we build on these theories by inferring that in addition, the possibility of failure to cold-weld the head and taper together on insertion may have led to excessive trunnion wear. This theory is further strengthened by the fact that there does not exist an accurate method to assess the cold-weld of the head on the stem at final hardware insertion apart from an intraoperative shuck test. With such uncertainty around patients at greatest risk of trunnion wear, failure, and trunnionosis, enhanced guidelines around recommended postoperative THA follow-up protocols are required [5]. Currently, there is a paucity of information available to guide postoperative follow-up frequency and duration after THA. There are currently no established guidelines from the Canadian Orthopedic Association (COA) with respect to long-term follow-up of THA patients. A broader search of guidelines used amongst orthopedic societies worldwide yielded recommendations from the British Hip Society, which include follow-up with radiographic assessment annually for the first five years, biennially for the following five, and triennially thereafter. Additionally, a survey of current practices by the American Association of Hip and Knee Surgeons in 2003 illustrated that most surgeons recommend annual follow-up for the first five years post THA, biennial follow-up for the following five years, and either annually or biennially afterwards [12]. In the case illustrated here, follow-up was limited to only one-year post-operatively after which time the patient was lost to follow-up. Given that trunnion wear with the Accolade total hip arthroplasty system is a rare complication, we do not currently have a strong understanding of such event rates. Ultimately, it will be important for future research to explore the rates of trunnion wear using the Accolade total hip arthroplasty system, as well as time to failure and outcomes related to early versus late intervention in this specific patient population.
